# Supplementary material for: The Fab region of IgG impairs the internalization pathway of FcRn upon Fc engagement
Source: Nat Commun. 2022 Oct 14;13:6073. doi: 10.1038/s41467-022-33764-1 (PMC9568614; doi:10.1038/s41467-022-33764-1)
Supplement: Supplementary file 1 — Supplementary Information [file 41467_2022_33764_MOESM1_ESM.pdf]

The Fab region of IgG impairs the internalization pathway of FcRn upon Fc engagement

Maximilian Brinkhaus, Erwin Pannecoucke, Elvera J. van der Kooi, Arthur E. H. Bentlage, Ninotska I. L. Derksen, Julie Andries, Bianca Balbino, Magdalena Sips, Peter Ulrichts, Peter Verheesen, Hans de Haard, Theo Rispens, Savvas N. Savvides, Gestur Vidarsson

# - Supplementary Information -

Supplementary Figure 1

a

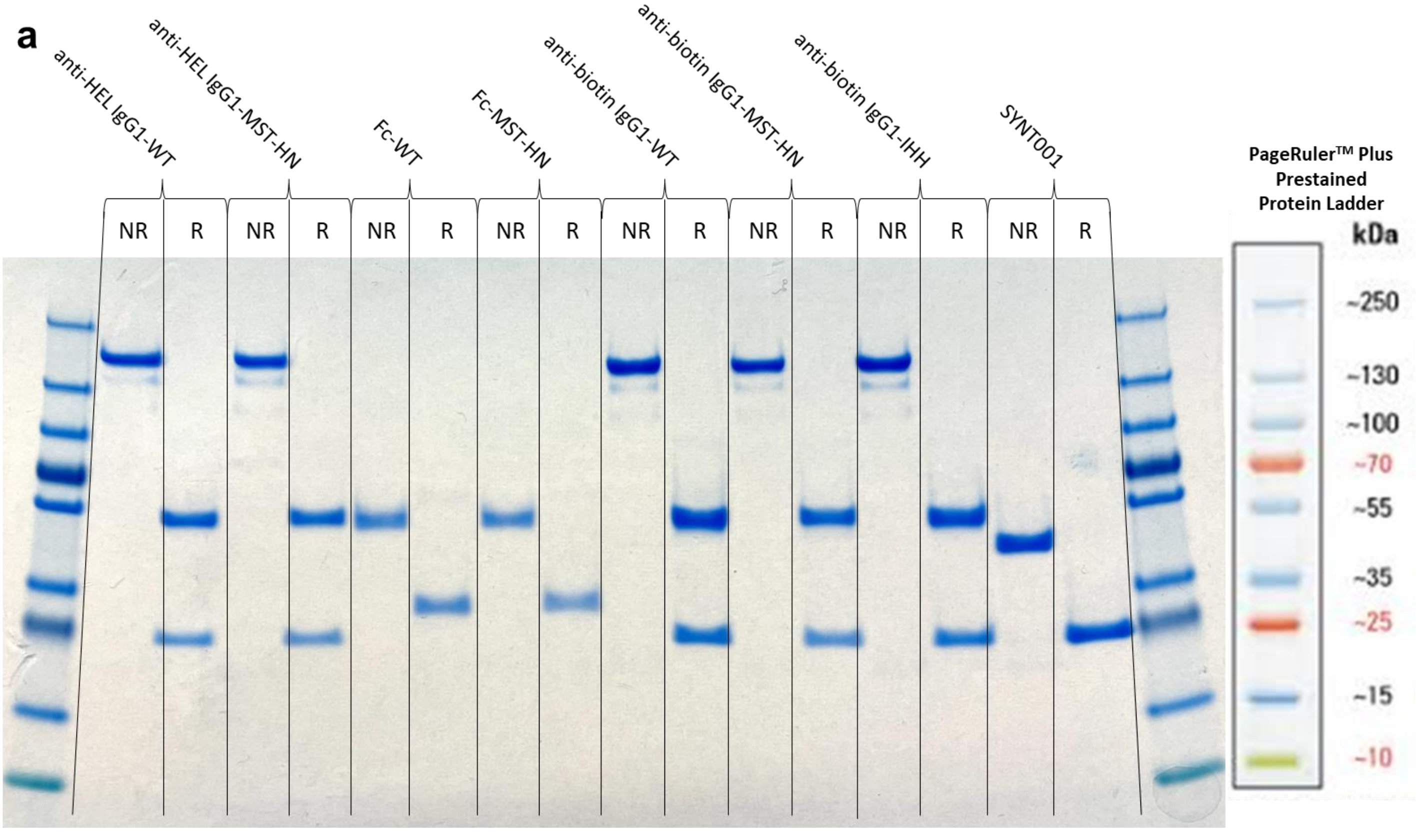

b

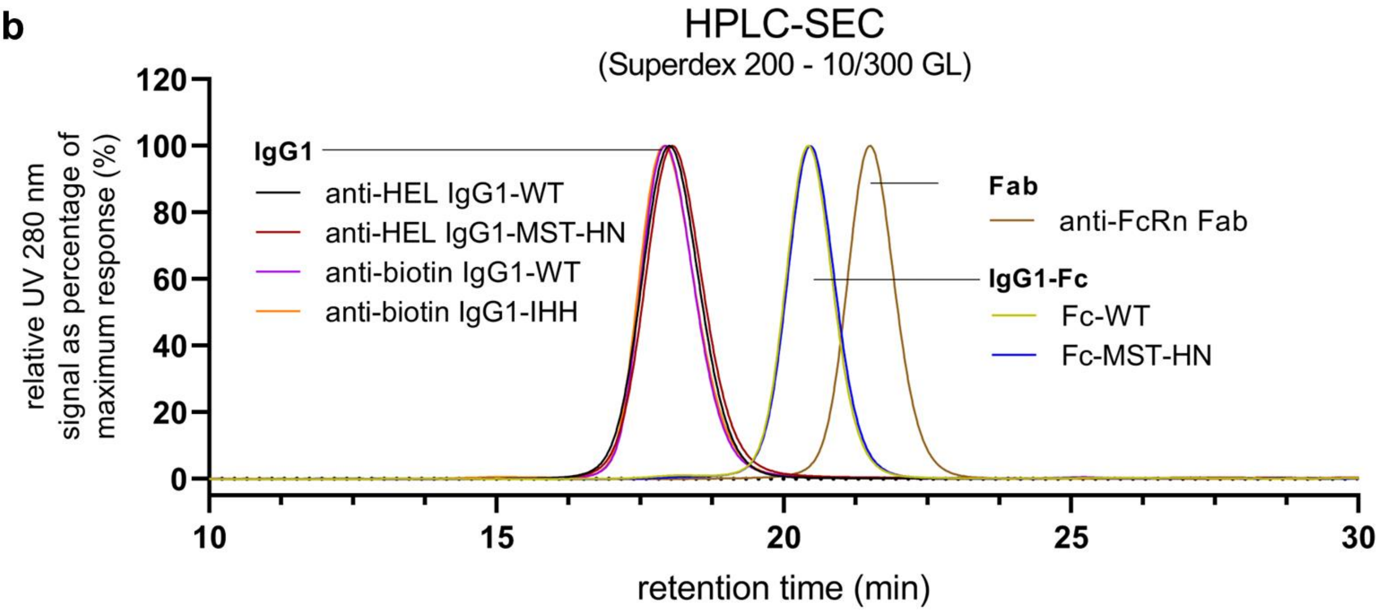

Supplementary Fig. 1. Biochemical quality control of antibody(fragments) used in the study. (a) SDS-PAGE of antibody(fragments) under reducing (R) and non-reducing (NR) conditions. (b) HPLC-SEC chromatograms of the antibody(fragments) as relative UV280 nm signal normalized to maximum response. Chromatograms of the same molecule type strongly overlap. SDS-PAGE and HPLC-SEC have been performed once.

Supplementary Figure 2

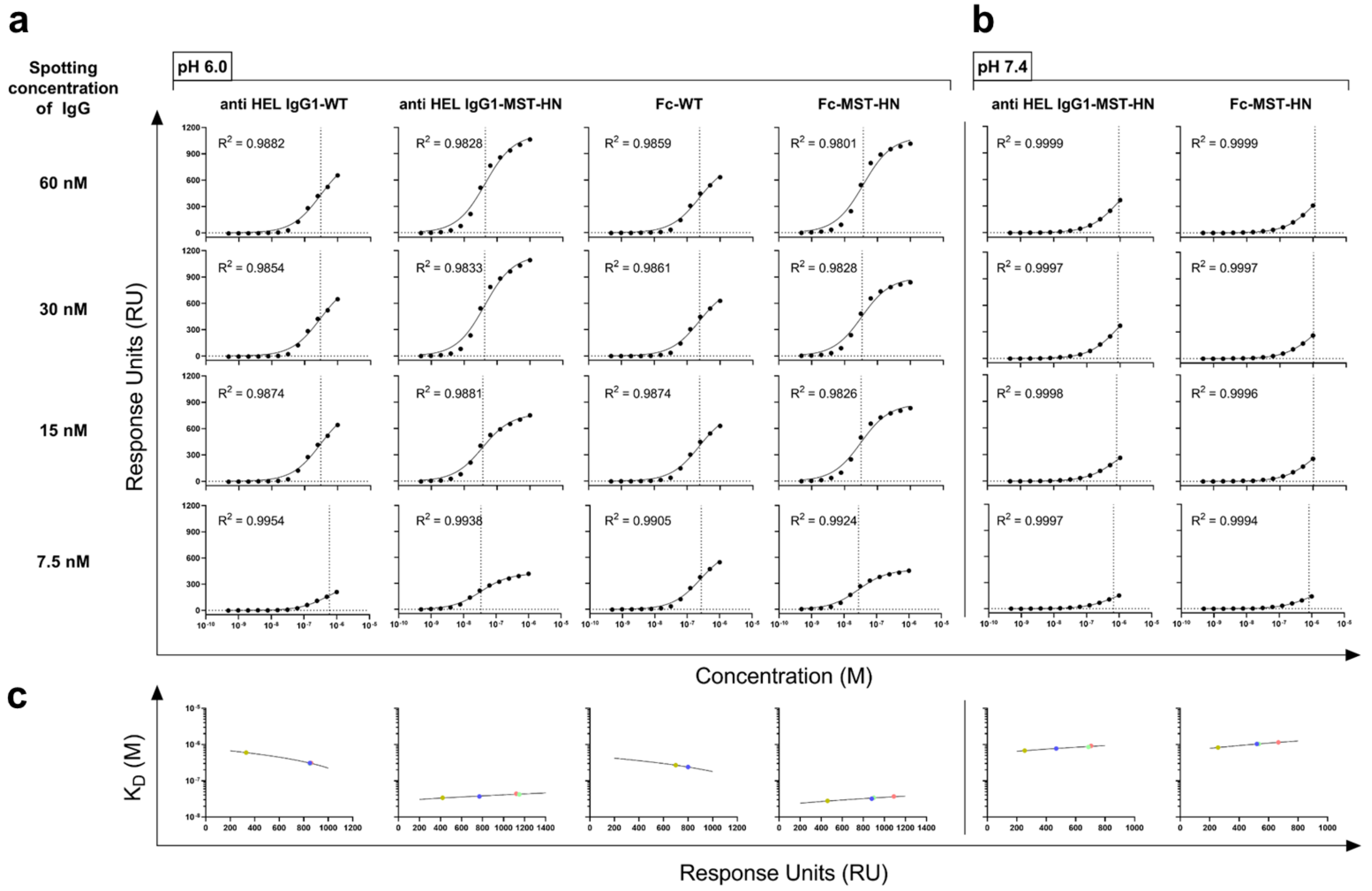

Supplementary Fig. 2. Affinity plots obtained from equilibrium analysis for each spotting concentration as used for affinity calculations by fitting a 1:1 Langmuir model. (a) Affinity plots at pH 6.0, (b) at pH 7.4 were used to calculate K_D and R_{max} values which were used to interpolate a K_D at R_{max}=700. RUs were determined at 360 sec at the end of the association time (Fig. 1a). Representative data of three independent experiments. Estimation for goodness of fit by fitting a non-linear regression (one site - specific binding) with a confidence interval of 95%. (c) K_D and R_{max} values obtained from affinity plots in (a) and (b) plotted for an interpolation of the K_D to an Rmax of 700.

Supplementary Figure 3

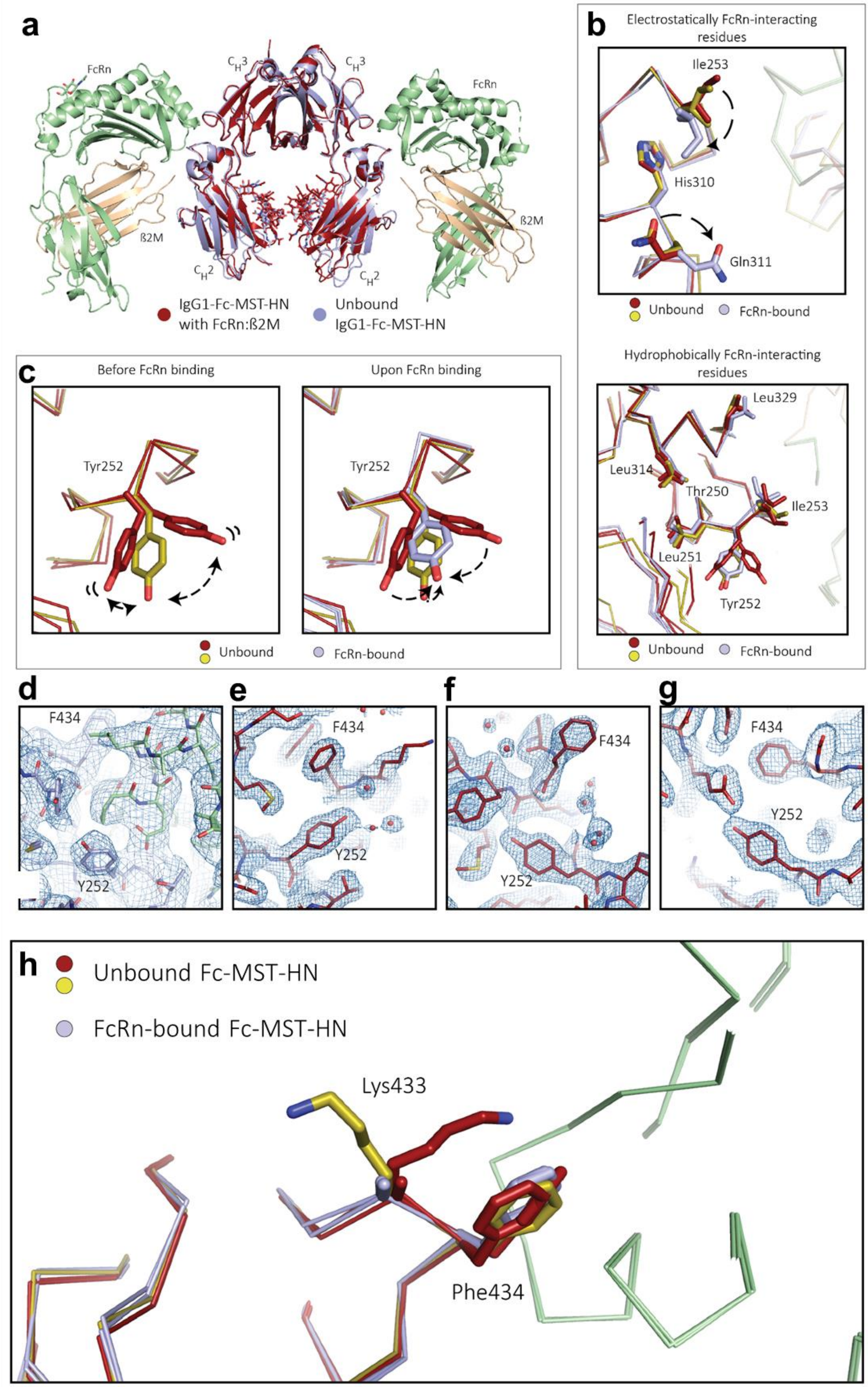

Supplementary Fig. 3. Structural comparison of unbound and FcRn-bound Fc-MST-HN. (a) Superposition of the crystallographic models of complexes between FcRn:ß2M: hFc-MST-HN (respectively green, orange and red) and unbound Fc-MST-HN (blue). (b) Detailed view of the amino acids that electrostatically and hydrophobically contribute to the binding of FcRn. (c) Detailed view demonstrating how FcRn-binding restricts the conformational freedom of Tyr252. (d) Fc-MST-HN in complex with FcRn and (e) chain A, (f) chain B and (g) chain C of unbound Fc-MST-HN. 2Fo-Fc difference electron density map is displayed at 1 r.m.s.d. and carved at 2 Å. (h) Detailed view of of Lys433 of Fc-MST-HN. One crystal diffracted to the reported resolution.

Supplementary Figure 4

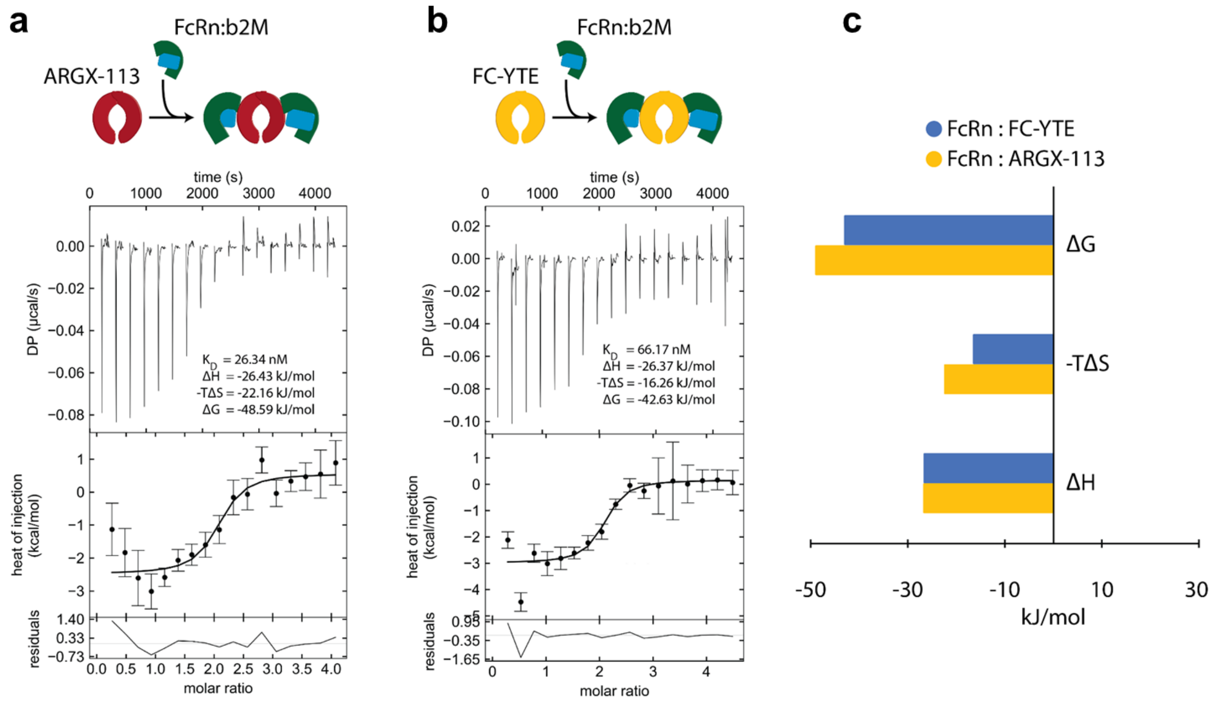

Supplementary Fig. 4. Thermograms of the interactions between FcRn:ß2M and (a) Fc-MST-HN or (b) FC-YTE, overlaid with the thermodynamic parameters. (c) Comparison of the thermodynamic footprints of the interactions, demonstrating that a reduction in entropic penalty (-TΔS) is at the root of the higher affinity of Fc-MST-HN for FcRn:ß2M, as measured by a lowered ΔG. For panel a, each kinetic parameter is the average of the individual fitted values which, consequently, breaks the logarithmic relationship between ΔG and the K_D. No meaningful difference could be observed in the enthalpic component (ΔH). The error bars in (a) and (b) indicate estimated errors of the integrated isotherm data point. This experiment was performed once for Fc-YTE and four times for ARGX-113 (Fc-MST-HN).

Supplementary Figure 5

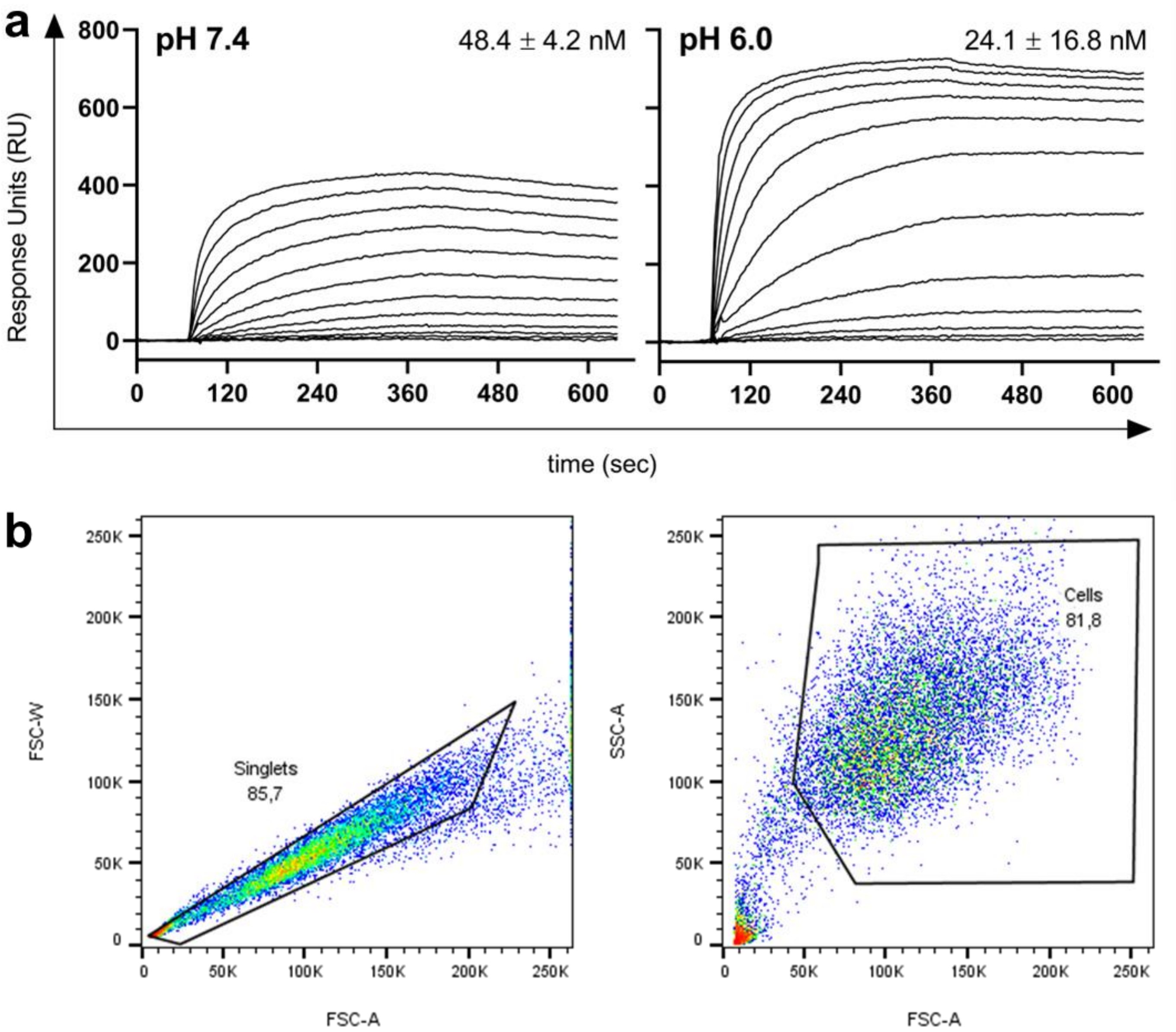

Supplementary Fig. 5. Characterization of Binding of anti-FcRn Fab to human FcRn at pH 6.0 and 7.4 in SPR and general gating strategy for flow cytometry experiments. (a) Human FcRn was titrated over anti-FcRn Fab at pH 6.0 and pH 7.4, which was randomly spotted on a sensor. K_D values were calculated by performing an equilibrium analysis and fitting a Langmuir 1:1 binding model. K_D values are averaged of three independent experiments and are presented as mean values with standard deviations. No statistical analysis was performed. (b) General gating strategy for flow cytometry-based experiments with HEK-FcRn-GFP cells.

Supplementary Figure 6

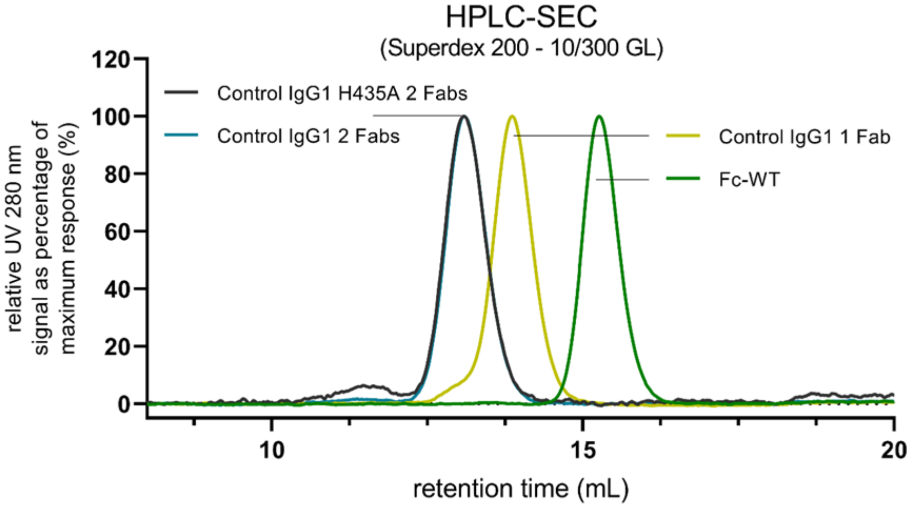

Supplementary Fig. 6. Integrity and size confirmation of non-FcRn modified IgG1-Fc backbones in HPLC-SEC HPLC-SEC chromatograms of the non-FcRn modified Control IgG variants as relative UV280 nm signal normalized to maximum response. Chromatograms of the same molecule type strongly overlap. This experiment was performed once.

Supplementary Figure 7

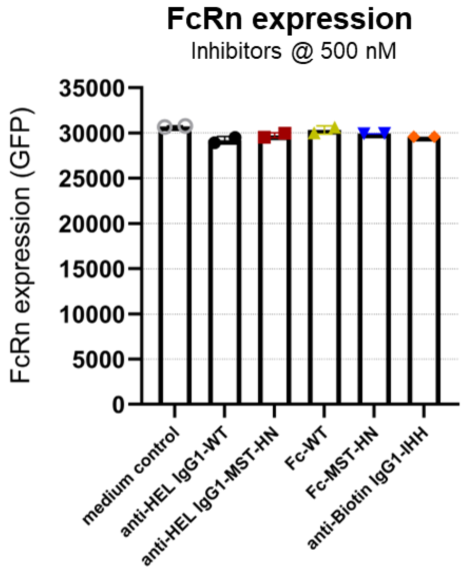

Supplementary Fig. 7. FcRn expression is not influenced by treatment of cells with inhibitors. FcRn-GFP signal of HEK-FcRn-GFP cells loaded with inhibitors at 500 nM in comparison to medium control. This experiment was performed once, the data represents aggregated data from a technical replicate presented as mean values. Error bars indicate standard deviations. No statistical analysis was performed.

Supplementary Figure 8

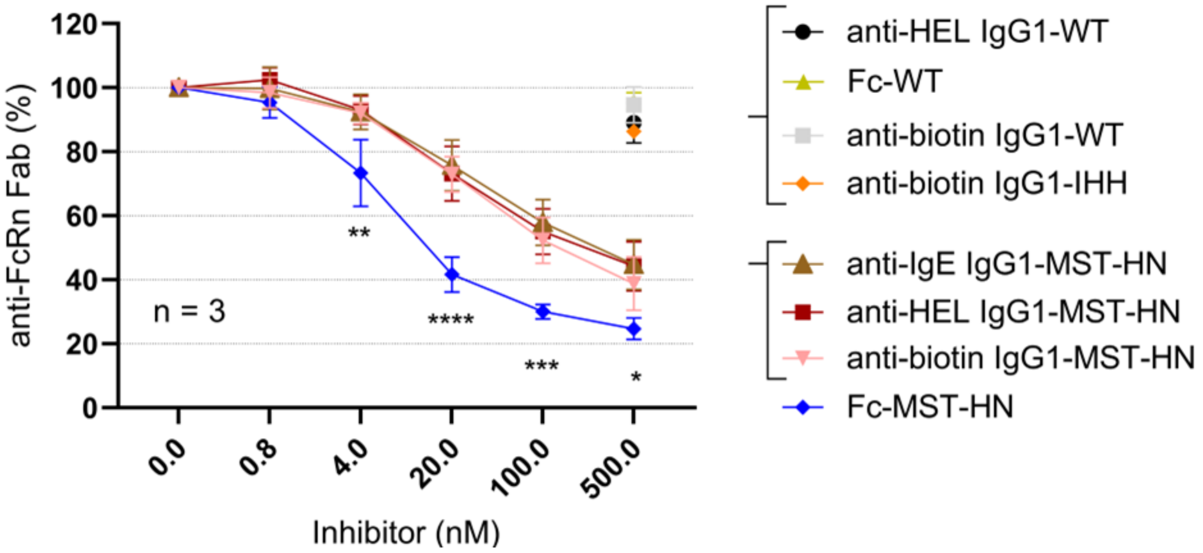

Supplementary Fig. 8. Fc-MST-HN shows higher levels of intracellular-FcRn occupancy compared to IgG1-MST-HN with different specificities. Anti-FcRn Fab-AF650 staining of cells plotted as percent gMFI in the presence of indicated inhibitors compared to untreated cells. Data represent aggregated data presented as mean values from three independent experiments. Errors bars indicate standard deviations. Statistical analysis was performed using a 2-way ANOVA (Sidak's multiple-comparisons test) and statistically significant differences are indicated by asterisks * < 0.05, ** <0.01, ***< 0.001, **** < 0.0001.

Supplementary Table 1

Table S1. Crystallographic data and refinement statistics

Protein complex	IgG1-Fc-MST-HN	IgG1-Fc-MST-HN : FcRn : ß2M
Crystallization		
PDB code	7Q3P	7Q15
Crystallization condition	0.2 M $CaCl_2$<br>20% w/v PEG 3350<br>pH 5.1	0.1 M Sodium Cacodylate pH 7<br>40% v/v MPD<br>3% w/v PEG 8000
cryoprotectant	20 % ethylene glycol	-
Data collection		
Beamline [a]	P14 (PETRAIII, EMBL Hamburg)	PROXIMA-2A (SOLEIL, France)
Wavelenght (Å) [a]	0.9763	0.89
Detector [a]	Dectris Eiger 16M	EIGER X 9M
Space group [a]	C 2	P 1 2_1 1
a, b, c (Å) [a]	96.408, 87.935, 106.093	86.429, 53.107, 195.745
α, β, γ (Å) [a]	90, 114.041, 90	90, 90.184, 90
Resolution (Å) [a]	46.61 - 2.097 (2.172 - 2.097) [a]	43.27 - 3.301 (3.419 - 3.301) [b]
Total reflections	330083 (30417) [a]	65905 (3341) [b]
Unique reflections	46871 (4414) [a]	21510 (1073) [b]
Multiplicity	7.0 (6.9) [a]	3.1 (3.1) [b]
Spherical completeness (%)	98.76 (93.89) [a]	68.2 (14.3) [b]
Ellipsoidal completeness (%)	*n.a.*	90.4 (62.2) [b]
Mean I/σ	12.42 (1.09) [a]	4.2 (1.1) [b]
Wilson B-factor (Å²)	40.42 [c]	60.88 [b]
R-meas (%)	12.34 (186.9) [a]	38.1 (156.0) [b]
CC1/2 (%)	99.88 (46.4) [a]	94.2 (26.5) [b]
Refinement [c]		
Resolution range (Å)	46.61 - 2.097	48.9 - 3.301
Reflections in refinement	46850 (4414)	18236 (135)
Reflections used for R-free	2343 (221)	1003 (12)
R-work	0.1872 [d]	0.2212 [e]
R-free	0.2185 [d]	0.2360 [e]
Non-hydrogen atoms	5664	8536
Macromolecular atoms	4975	8316
Ligand atoms	294	220
Solvent atoms	395	0
Protein residues	650	1139
RMS_{bounds} (Å)	0.009	0.01
RMS_{angles} (°)	1.56	1.57
Ramachandran favored (%)	99.19	96.69
Ramachandran allowed (%)	0.81	3.22
Ramachandran outliers (%)	0	0.09
Rotamer outliers (%)	0.71	0.64
Clashscore	7.26	18.80
Average B-factor (Å²)	50.88	57.50
Macromolecules	49.72	56.03
Ligands	69.30	113.27
Solvent atoms	51.70	n.a.
TLS groups	16	6

Values in parentheses correspond to the highest-resolution shell. [a] Values reported by XDS. [b] Values reported by STARANISO anisotropy & Bayesian estimation server. [c] Values reported by Phenix. [d]. Final refinement was performed in Phenix 1.13-2998. [e] final refinement was performed by BUSTER 2.10.3
