# Supplementary material for: The Fab region of IgG impairs the internalization pathway of FcRn upon Fc engagement
Source: Nat Commun. 2022 Oct 14;13:6073. doi: 10.1038/s41467-022-33764-1 (PMC9568614; doi:10.1038/s41467-022-33764-1)
Supplement: Supplementary file 3 — Reporting Summary [file 41467_2022_33764_MOESM3_ESM.pdf]

nature portfolio

Corresponding author(s): Gestur Vidarsson

Last updated by author(s): Sep 2, 2022

Reporting Summary

Nature Portfolio wishes to improve the reproducibility of the work that we publish. This form provides structure for consistency and transparency in reporting. For further information on Nature Portfolio policies, see our Editorial Policies and the Editorial Policy Checklist.

Statistics

For all statistical analyses, confirm that the following items are present in the figure legend, table legend, main text, or Methods section.

n/a	Confirmed	
☐	☒	The exact sample size (*n*) for each experimental group/condition, given as a discrete number and unit of measurement
☐	☒	A statement on whether measurements were taken from distinct samples or whether the same sample was measured repeatedly
☐	☒	The statistical test(s) used AND whether they are one- or two-sided<br>*Only common tests should be described solely by name; describe more complex techniques in the Methods section.*
☐	☒	A description of all covariates tested
☐	☒	A description of any assumptions or corrections, such as tests of normality and adjustment for multiple comparisons
☐	☒	A full description of the statistical parameters including central tendency (e.g. means) or other basic estimates (e.g. regression coefficient) AND variation (e.g. standard deviation) or associated estimates of uncertainty (e.g. confidence intervals)
☐	☒	For null hypothesis testing, the test statistic (e.g. *F*, *t*, *r*) with confidence intervals, effect sizes, degrees of freedom and *P* value noted<br>*Give P values as exact values whenever suitable.*
☐	☒	For Bayesian analysis, information on the choice of priors and Markov chain Monte Carlo settings
☐	☒	For hierarchical and complex designs, identification of the appropriate level for tests and full reporting of outcomes
☒	☐	Estimates of effect sizes (e.g. Cohen's *d*, Pearson's *r*), indicating how they were calculated

Our web collection on statistics for biologists contains articles on many of the points above.

Software and code

Policy information about availability of computer code

Data collection	We have not used customized code for data collection. Flow cytometry data was aquired using BD FACS Diva Software 8.0.1, IBIS iSPR Version 6.19.3.17. was used to aquire SPR data.
Data analysis	Srubber 2, Sprint 1.9.4.4, FlowJo 10.7.1, BD FACS Diva , GraphPad Prism 8, MS Office Excel Version 2205 (build 15225.20394), SAS Viya release V.03.04

For manuscripts utilizing custom algorithms or software that are central to the research but not yet described in published literature, software must be made available to editors and reviewers. We strongly encourage code deposition in a community repository (e.g. GitHub). See the Nature Portfolio guidelines for submitting code & software for further information.

Data

Policy information about availability of data

All manuscripts must include a data availability statement. This statement should provide the following information, where applicable:

- Accession codes, unique identifiers, or web links for publicly available datasets
- A description of any restrictions on data availability
- For clinical datasets or third party data, please ensure that the statement adheres to our policy

The datasets generatred during this study and/or analysis during the currect study are available from the corresponding author on reasonable request. The data presented in the main and supplementary figures are provided in the Source Data file.

Human research participants

Policy information about studies involving human research participants and Sex and Gender in Research.

Reporting on sex and gender	Not applicable
Population characteristics	Not applicable
Recruitment	Not applicable
Ethics oversight	Not applicable

Note that full information on the approval of the study protocol must also be provided in the manuscript.

Field-specific reporting

Please select the one below that is the best fit for your research. If you are not sure, read the appropriate sections before making your selection.

☒ Life sciences ☐ Behavioural & social sciences ☐ Ecological, evolutionary & environmental sciences

For a reference copy of the document with all sections, see nature.com/documents/nr-reporting-summary-flat.pdf

Life sciences study design

All studies must disclose on these points even when the disclosure is negative.

Sample size

We have not predetermined the sample size. Every in vitro experiment was performed in duplicate and repeated 3 to 5 times, if not indicated differently. The cynomolgus monkey study was limited to two animals per group.

Data exclusions

No data has been excluded.

Replication

Figure 1:
SPR (a+b): 3 independent experiments (all replicates were successful)
Affinity chromatography (c): 2 independent experiments (all replicates were successful)

Figure 2:
More than 100 crystals were successfully generated, however, only one crystal diffracted to the reported resolution.

Figure 3:
Surface competition assay (a+b): 3 independent experiments (all replicates were successful)
Surface competition assay (c): 5 independent experiments (all replicates were successful)

Figure 4:
Intracellular FcRn occupancy (a+b): 3 independent experiments (all replicates were successful)
Intracellular accumulation (c): 3 independent experiments (all replicates were successful)

Figure 5:
Recycling assay controls (a): 4 independent experiments (all replicates were successful)
Recycling assay with inhibitors (b): 3 independent experiments (all replicates were successful)
Cynomolgus monkey experiment (c): 2 animals per group

Supplementary Figure 1:
SDS-PAGE (a): was performed once
HPLC (b): was performed once

Supplementary Figure 2:
Relates to Figure 1 (a+b): 3 independent experiments

Supplementary Figure 3:
More than 100 crystals were successfully generated, however, only one crystal diffracted to the reported resolution.

Supplementary Figure 4:
ITC: (a) 4 independent experiments, (b) one expeirment (all experiments were successful)

Supplementary Figure 5:
SPR (a): 3 independent experiments
Gating strategy (b): representative of all flow cytometry based assays

Supplementary Figure 6:
HPLC: The experiment was performed once.

Supplementary Figure 7:
FcRn expression: The experiment was performed once.

Supplementary Figure 8:
FcRn occupancy: 3 independent experiments (all replicates were succesful)

Randomization: Sample sizes and types did not warrant randomization. We used only cell line- and antibody-based samples comparing equal numbers of identical cells with different test items. Cynomolgus monkeys were randomly assigned to study groups.

Blinding: No samples were used warranting blinding of experimentators or data analysis.

Reporting for specific materials, systems and methods

We require information from authors about some types of materials, experimental systems and methods used in many studies. Here, indicate whether each material, system or method listed is relevant to your study. If you are not sure if a list item applies to your research, read the appropriate section before selecting a response.

Materials & experimental systems

n/a	Involved in the study	
☐	☒	Antibodies
☐	☒	Eukaryotic cell lines
☒	☐	Palaeontology and archaeology
☐	☒	Animals and other organisms
☒	☐	Clinical data
☒	☐	Dual use research of concern

Methods

n/a	Involved in the study	
☒	☐	ChIP-seq
☐	☒	Flow cytometry
☒	☐	MRI-based neuroimaging

Antibodies

Antibodies used: anti-human IgG-Fc HRP (Southern Biotech), Clone JDC-10, Cat. No. 9040-05

Validation: Most antibodies or fragments thereof used in this study were generated by ourselves or external companies for this project, and their validations were part of this peer review. Sequences of all these antibodies are available from patents and pubicly available sequences described in the Methods. The exception is the commercially available anti-human IgG-Fc HRP (Southern Biotech), Clone JDC-10, Cat. No. 9040-05 that has been validated by the supplier and was found to specifically bind to all human IgG subclasses, but not human IgA or IgM.

Eukaryotic cell lines

Policy information about cell lines and Sex and Gender in Research

Cell line source(s): HEK293 cells (Cell Line Service = CLS; product number 3000192) were used to generate HEK-FcRn-GFP cells. The HEK293 cells were obtained from CLS. HEK293F cells were obtained from Thermo Fisher Scientific.

Authentication: HEK293 cells were authenticated by CLS by STR analysis. After in-house modification (stable transfection of FcRn-GFP), cells were not authenticated again, but functionally characterized regarding their FcRn-GFP expression.

Mycoplasma contamination: Cells were purchased as mycoplasma negative cells, but not regularly tested for mycoplasma contamination.

Commonly misidentified lines (See ICLAC register): HEK293; cells were purchased as authenticated and thereafter stably transfected with FcRn-GFP. The stably transfected cell line was used for this study and has been functionally characterized regarding its FcRn expression.

Animals and other research organisms

Policy information about studies involving animals; ARRIVE guidelines recommended for reporting animal research, and Sex and Gender in Research

Laboratory animals: Female cynomolgus monkeys (Macaca fascularis) of 3.1 to 3.5 years (at first dosing)

Wild animals	The study did not involve wild animals.
Reporting on sex	Female cynomolgus monkeys were used for the study, however, the (pre)clinical data generated does not suggest differences between female or male.
Field-collected samples	No field-collected samples were used in this study.
Ethics oversight	Niedersaechsisches Landesamt fuer Verbraucherschutz und Lebensmittelsicherheit, Wardenburg, Germany (ethics committee state of lower saxony, under the german animal welfare law)

Note that full information on the approval of the study protocol must also be provided in the manuscript.

Flow Cytometry

Plots

Confirm that:

☒ The axis labels state the marker and fluorochrome used (e.g. CD4-FITC).

☒ The axis scales are clearly visible. Include numbers along axes only for bottom left plot of group (a 'group' is an analysis of identical markers).

☒ All plots are contour plots with outliers or pseudocolor plots.

☒ A numerical value for number of cells or percentage (with statistics) is provided.

Methodology

Sample preparation	Only HEK-FcRn-GFP cells were used for flow cytometry experiments originating from an adherent culture, which was passaged by trypsinization when 80 - 90% confluence was reached.
Instrument	BD FACS Canto-II Cell Analyzer (BD 371 Biosciences), BD LSR-II (BD Biosciences), BD LSRFortessa (BD Biosciences)
Software	FlowJo 10.7.1, FACS Diva Software (BD Biosciences)
Cell population abundance	Only a cell line was used for flow cytometry experiments, and therefore only debris and doublets were gated out. A representative gating strategy is included in Supplementary Figure 5b. A minimal of 5000 events, but mostly 10000 events, if possible, were recorded.
Gating strategy	Singlets were gated (FSC-A/FSC-W), then the cell population was gated (gating out debris) (FSC-A/ SSC-A).

☒ Tick this box to confirm that a figure exemplifying the gating strategy is provided in the Supplementary Information.
